# Aging Ni(OH)_2_ on 3C-SiC Photoanodes to Achieve a High Photovoltage of 1.1 V and Enhanced Stability for Solar Water Splitting in Strongly Alkaline Solutions

**DOI:** 10.1021/acsami.4c11809

**Published:** 2024-09-17

**Authors:** Yuanju Qu, Valdas Jokubavicius, Duc Quang Hoang, Xianjie Liu, Mats Fahlman, Ivan G. Ivanov, Rositsa Yakimova, Jianwu Sun

**Affiliations:** † Department of Physics, Chemistry and Biology (IFM), Linköping University, Linköping SE-58183, Sweden; ‡ Laboratory of Organic Electronics, Department of Science and Technology, Linköping University, Norrköping 60174, Sweden

**Keywords:** cubic silicon carbide (3C-SiC), solar water splitting, solar-to-hydrogen conversion, photovoltage, aging of Ni(OH)_2_

## Abstract

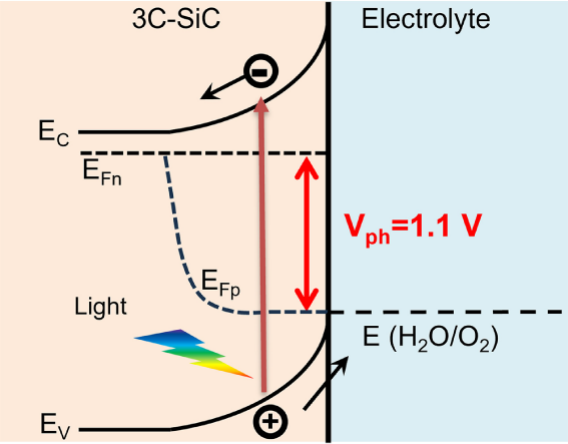

Photoelectrochemical (PEC) water splitting is a promising approach to directly convert solar energy to renewable and storable hydrogen. However, the very low photovoltage and serious corrosion of semiconductor photoelectrodes in strongly acidic or alkaline electrolytes needed for water splitting severely impede the practical application of this technology. In this work, we demonstrate a facile approach to fabricate a high-photovoltage, stable photoanode by depositing Ni(OH)_2_ cocatalyst on cubic silicon carbide (3C-SiC), followed by aging in 1.0 M NaOH at room temperature for 40 h without applying electrochemical bias. The aged 3C-SiC/Ni(OH)_2_ photoanode achieves a record-high photovoltage of 1.10 V, an ultralow onset potential of 0.10 V vs the reversible hydrogen electrode, and enhanced stability for PEC water splitting in the strongly alkaline solution (pH = 13.6). This aged photoanode also exhibits excellent in-air stability, demonstrating identical PEC water-splitting performance for more than 400 days. We find that the aged Ni(OH)_2_ dramatically promotes the hole transport at the photoanode/electrolyte interface, thus significantly enhancing the photovoltage and overall PEC performance. Furthermore, the oxygen evolution reaction (OER) activity and the phase transitions of the Ni(OH)_2_ electrocatalyst before and after aging are systematically investigated. We find that the aging process is critical for the formation of the relatively stable and highly active Fe-doped β-NiOOH, which accounts for the enhanced OER activity and stability of the PEC water splitting. This work provides a simple and effective approach to fabricate high-photovoltage and stable photoanodes, bringing new premise toward solar fuel development.

## Introduction

Photoelectrochemical (PEC) water splitting has long been considered an ideal approach to directly convert the most abundant but intermittent solar energy into renewable and storable hydrogen, which is one of the pillar stones in pursuit of an energy-sustainable society to address current environmental issues and energy crisis.^1−3^ For the PEC water splitting cell, the semiconductor photoelectrode is the key component that absorbs sunlight energy and generates the photovoltage driving solar water splitting.^4,5^ Since Fujishima and Honda’s pioneering work using TiO_2_ for PEC water splitting,^6^ great efforts have been made to explore various photoelectrodes including Si, WO_3_, α-Fe_2_O_3_, BiVO_4_, Ta_3_N_5_, and IIV–V semiconductors.^7−9^ However, the very low photovoltages reported so far and the serious corrosion of semiconductor photoelectrodes in strongly acidic or alkaline electrolytes needed for water splitting severely impede the practical application of this technology.

To drive solar water splitting, a photovoltage exceeding 1.23 V is generally required. Although photovoltaic materials exhibit large photovoltages that are approaching their Shockley–Queisser (S-Q) limits,^10,11^ the PEC photoanodes and cathodes including their buried junctions reported so far show very low photovoltage (less than 1 V) regardless of their bandgaps (Table S1).^11^ Si, the most studied photovoltaic material, exhibited a photovoltage of 0.72 V in solar cells, approaching its (S-Q) limit.^10,11^ However, Si photoanodes with buried junctions generally show large onset potentials 1.0–1.2 V versus the reversible hydrogen electrode (V_RHE_) and low photovoltages (<0.60 V) for PEC water splitting (Table S1).^11^ With a suitable bandgap (E_g_) of 2.0 eV, α-Fe_2_O_3_ has been widely studied as a photoanode, but it usually shows large onset potentials of 0.8–1.0 V_RHE_ and small photovoltages (typically less than 0.4 V). The benchmark photovoltage of 0.8 V was reported for the α-Fe_2_O_3_ photoanode using a regrowth method.^12^ Although BiVO_4_ (E_g_ ∼ 2.4 eV) showed relatively small onset potentials of 0.2–0.4 V_RHE_ and relatively large photovoltages of 0.8–1.0 V, it mainly operates in neutral electrolytes (pH ∼ 7) because it suffers from dissolution and corrosion in strong basic and acidic solutions. Up to date, the practical water-splitting devices can only be constructed in either alkaline or acidic media.^13^ Such electrolytes have the benefits of high conductivity and negligible pH gradient near the electrode under operating conditions and allow the use of gas-impermeable, ion exchange membranes for constructing safe water-splitting cells. However, many semiconductor photoanodes of interest, including Si, BiVO_4_, Ta_3_N_5_, and III–V semiconductors, are easily dissolved and/or photocorroded in strongly alkaline or acidic electrolytes, limiting their practical applications for PEC water splitting.^14−19^

During past decades, significant efforts have been dedicated to engineering semiconductor photoelectrodes with protective coatings and catalytic overlayers to promote stability (Table S1).^15−23^ Metal oxides such as TiO_2_ deposited by atomic layer deposition (ALD) have been widely used to protect photoelectrodes against corrosion. Yu et al. reported that black silicon photoanode with an ALD-TiO_2_ protective layer showed a 19% loss of its initial photocurrent after 4 h of continuous operation in 1.0 M NaOH (pH = 13.6), while the unprotected counterparts exhibited a 77% loss of the photocurrent after 3 h of operation.^20^ Lichterman et al. reported that the ALD protective layer of CoO_*x*_ enabled BiVO_4_ photoanodes to work in alkaline solution (pH 13) with ∼50% loss of the initial photocurrent after 1 h of operation.^21^ It was also reported that a polymer-coated BiVO_4_ photoanode exhibited improved stability, showing that 70% of its initial photocurrent remained after 6 h of operation in 0.1 M phosphate (pH = 12).^15^ Recently, Ta_3_N_5_ photoanodes gained intense research interest and showed a high photocurrent of 8–10 mA/cm^2^ at 1.23 V_RHE_.^22^ However, it showed large onset potentials (typically, 0.6–0.8 V_RHE_) and poor stability in alkaline solution (48% loss of its initial photocurrent after 80 min of operation in 1.0 M KOH.^23^ To date, it still remains very challenging to achieve an efficient photoelectrode with high photovoltage and long-term stability for PEC water splitting in harsh electrolytes.

Cubic silicon carbide (3C-SiC) has attracted much interest in solar water splitting due to its prominent material properties.^24−27^ 3C-SiC has a relatively small band gap of 2.36 eV, which is favorable for visible sunlight absorption. This bandgap is close to the ideal band gap (2.03 eV) of a single material for a maximum of the solar water splitting efficiency.^28^ In contrast to the extensively studied PEC semiconductors such as Si, Fe_2_O_3_, Cu_2_O, GaAs, and InP, the conduction and valence band positions of 3C-SiC ideally straddle the water redox potentials,^25^ enabling photogenerated carriers to gain enough energy to overcome the energetic barrier of PEC water splitting without applying an external bias. Moreover, compared to most semiconductors, 3C-SiC is highly stable in both strongly acid and alkaline solutions.^25^ However, despite great efforts made during the past decade, it is still very challenging to grow 3C-SiC. Recently, our group has shown that high-quality 3C-SiC films can be grown by the sublimation technique.^29−31^

In this work, we demonstrate a facile approach to fabricate a high-photovoltage, stable photoanode by depositing a Ni(OH)_2_ cocatalyst on 3C-SiC, followed by aging in 1.0 M NaOH at room temperature for 40 h without applying electrochemical bias. The aged 3C-SiC/Ni(OH)_2_ photoanode achieves a record-high photovoltage of 1.10 V and an ultralow onset potential of 0.10 V_RHE_, distinctly outperforming most of the reported photoanodes (Table S1). Moreover, this aged photoanode also exhibits excellent in-air stability (over 400 days) and outstanding operational stability for PEC water splitting in strongly alkaline solutions (pH = 13.6). The oxygen evolution reaction (OER) activity and the phase transitions of the Ni(OH)_2_ electrocatalyst before and after aging are systematically investigated. We find that the aging process is critical for the formation of the relatively stable and highly active Fe-doped β-NiOOH, which accounts for the enhanced OER activity and stability of the PEC water splitting.

## Result and Discussion

### 3C-SiC Photoanodes

**Figure 1:**
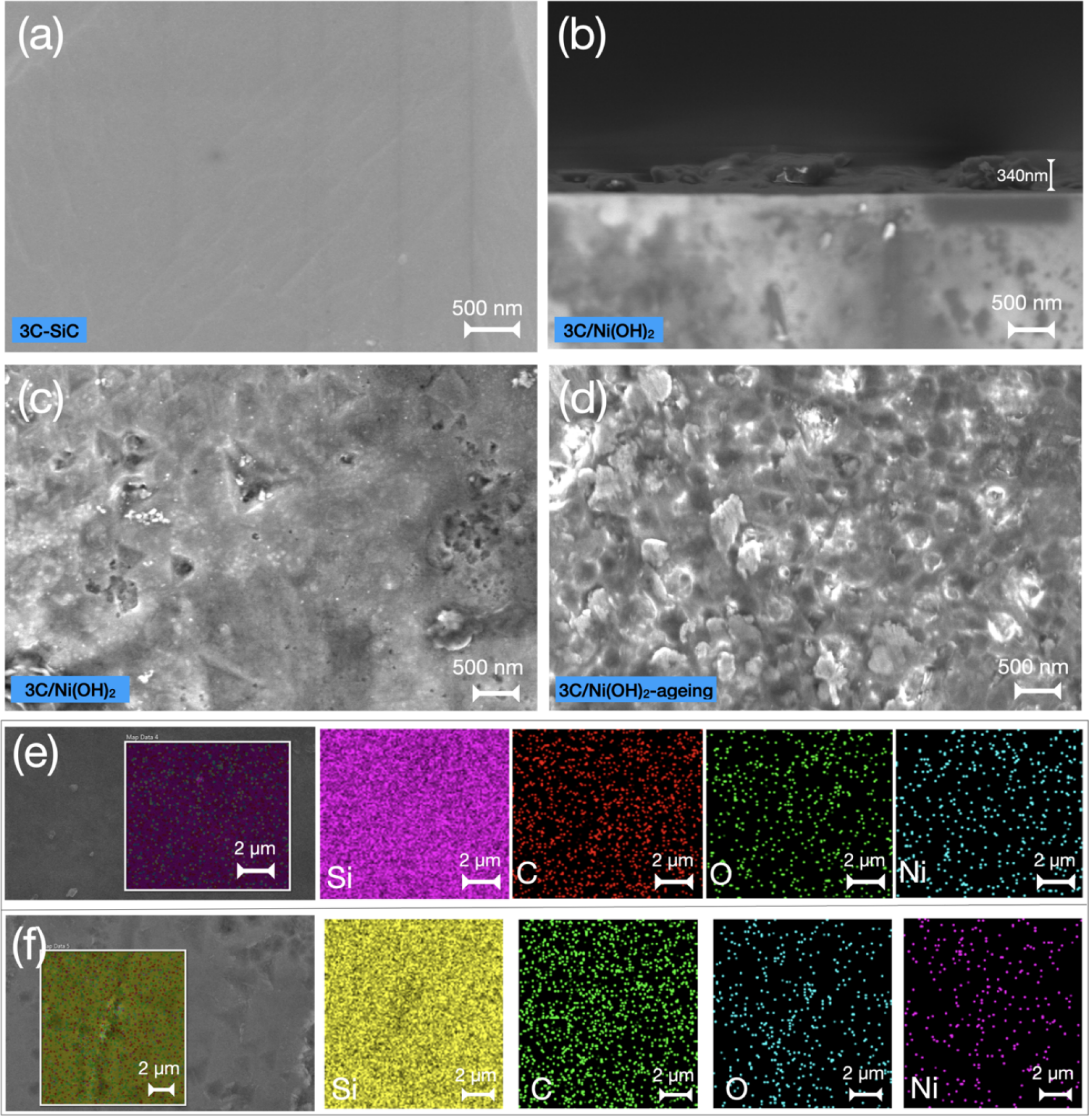
Top-view SEM images of (a) 3C-SiC, (c) 3C-SiC/Ni(OH)_2_, and (d) 3C-SiC/Ni(OH)_2_ after aging in 1.0 M NaOH solution for 40 h. Cross-sectional SEM image of (b) 3C-SiC/Ni(OH)_2_. EDX mapping of elements of (e) 3C-SiC/Ni(OH)_2_ and (f) 3C-SiC/Ni(OH)_2_-aging.

The 3C-SiC layers with a thickness of ∼300 μm were grown on 6H-SiC(0001) substrates by sublimation epitaxy.^29,31^ The absorption spectrum of 3C-SiC shows a sharp band-edge absorption, yielding a bandgap of 2.36 eV (Figure S1). The top-view scanning electron microscopy (SEM) image of the as-grown 3C-SiC exhibits a smooth surface (Figure 1a). A thin layer of Ni(OH)_2_ was deposited on the 3C-SiC surface (hereafter denoted as 3C/Ni(OH)_2_) by a facile hydrothermal method.^32^ The cross-sectional SEM image depicts the thickness of Ni(OH)_2_ as ∼340 nm (Figure 1b), and the top-view SEM image of 3C/Ni(OH)_2_ shows a dense layer of nanoparticles deposited on the 3C-SiC surface (Figures 1c and S2a). The energy dispersive X-ray spectroscopy (EDX) images of the top-view and cross-section of 3C/Ni(OH)_2_ clearly indicates that the deposited layer is composed of Ni and O elements (Figures 1e and S3). To investigate the aging effect of Ni(OH)_2_ on the PEC water-splitting performance, the prepared 3C/Ni(OH)_2_ was immersed in 1.0 M NaOH for 40 h at room temperature without applying electrochemical bias (hereafter denoted as 3C/Ni(OH)_2_-aging). After aging, the surface morphology of Ni(OH)_2_ is dramatically changed, showing arrays of large nanoparticle grains with the size of approximately 100–500 nm (Figures 1d and S2b). The EDX results confirm the presence of Ni and O elements in aged Ni(OH)_2_ on 3C-SiC (Figure 1f).

### PEC Performance of 3C-SiC Photoanodes

**Figure 2:**
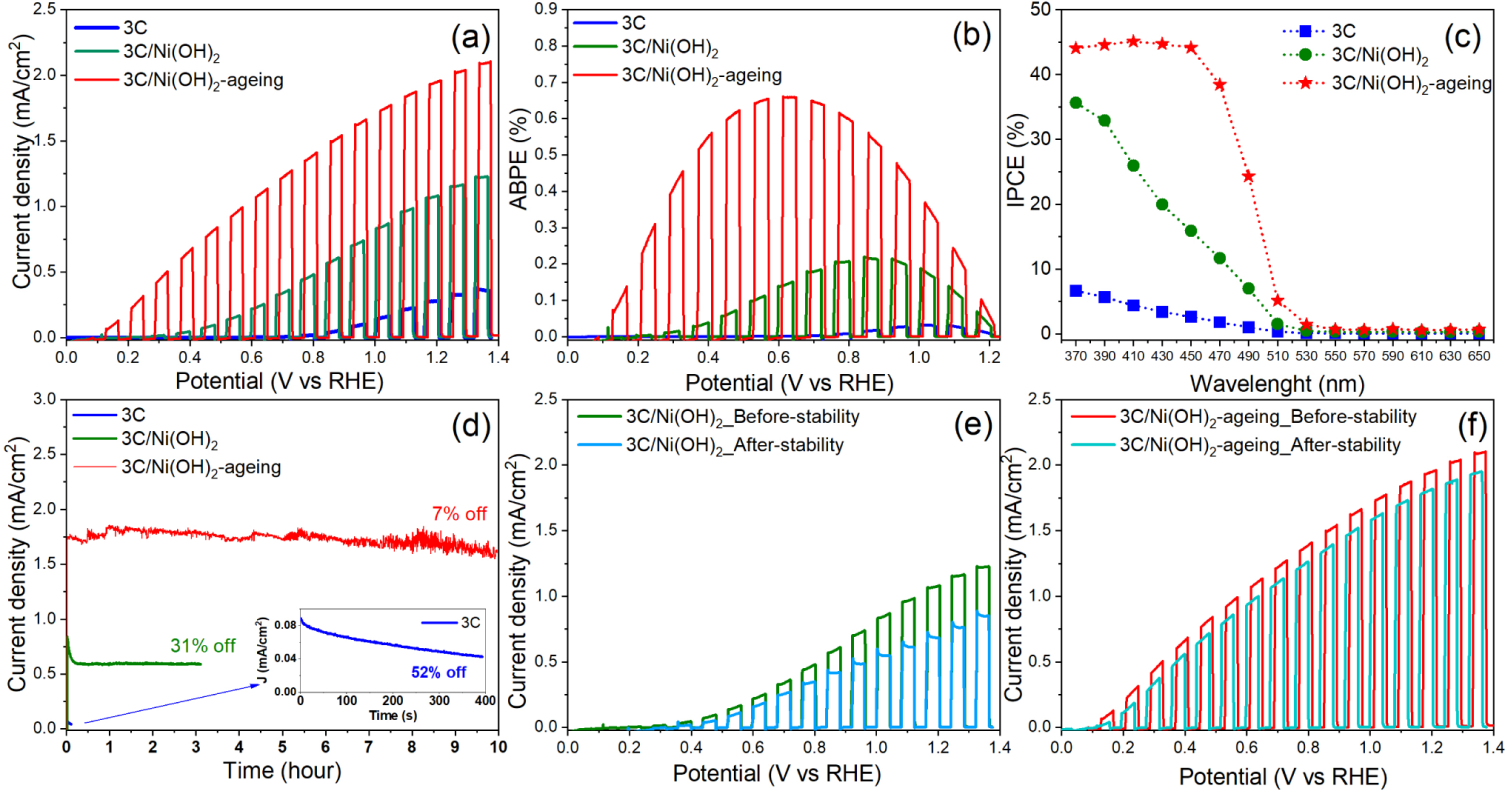
(a) Current density–potential curves and (b) ABPE of 3C-SiC, 3C-SiC/Ni(OH)_2_, and 3C-SiC/Ni(OH)_2_-aging photoanodes measured in 1.0 M NaOH electrolyte under chopped AM1.5G 100 mW/cm^2^ illumination, at a scan rate of 20 mV/s. (c) IPCE of the photoanodes measured in 1.0 M NaOH at 1.0 V_RHE_. (d) Measured chronoamperometry as stability tests of the three photoanodes in 1.0 M NaOH at 1.0 V_RHE_ under steady-state AM1.5G 100 mW/cm^2^ illumination, and the inset is the enlarged figure of stability test of 3C-SiC. *J*–*V* curves of (e) 3C-SiC/Ni(OH)_2_ and (f) 3C-SiC/Ni(OH)_2_-aging before and after the stability test.

The PEC water-splitting performance was evaluated in 1.0 M NaOH electrolyte under chopped AM1.5G at 100 mW cm^–2^ illumination. Figure 2a shows the photocurrent density–potential (*J*–*V*) curves of 3C-SiC, 3C/Ni(OH)_2_, and 3C/Ni(OH)_2_-aging photoanodes. With the deposition of Ni(OH)_2_ followed by aging, the 3C/Ni(OH)_2_-aging photoanode exhibits the highest photocurrent density of 2.01 mA/cm^2^ at 1.23 V versus the reversible hydrogen electrode (V_RHE_), distinctly outperforming the 3C/Ni(OH)_2_ (1.10 mA/cm^2^) and 3C-SiC (0.30 mA/cm^2^) at the same potential (Figure 2a). Notably, the 3C/Ni(OH)_2_-aging photoanode shows no photocurrent spikes (transient photocurrents when switching light on/off) even at a very low bias potential of 0.10 V_RHE_ under chopped light illumination (Figures 2a and S4c). In contrast, the 3C-SiC and 3C-SiC/Ni(OH)_2_ photoanodes exhibit transient photocurrents when switching the light on/off (Figure S4). Transient photocurrents are commonly observed for almost all studied photoelectrodes and are related to charge recombination via surface states of the photoelectrodes.^33−35^ Therefore, the absence of transient photocurrents at the 3C/Ni(OH)_2_-aging photoanode indicates that the aged Ni(OH)_2_ cocatalyst suppresses surface recombination and facilitates charge (photogenerated holes) transfer for water oxidation.

Moreover, the 3C/Ni(OH)_2_-aging photoanode exhibits a very low onset potential (*E*_onset_) of 0.10 V_RHE_, which is significantly reduced (cathodically shifted) compared to the onset potentials of 3C-SiC (*E*_onset_ = ∼ 0.60 V_RHE_) and 3C/Ni(OH)_2_ (*E*_onset_ = ∼ 0.20 V_RHE_), as shown in Figures 2a and S4. The low onset potential of 0.10 V_RHE_ suggests a high photovoltage of 1.13 V at the 3C/Ni(OH)_2_-aging photoanode, which is estimated by the difference between the onset potential and the Nernstian potential of water oxidation (1.23 V_RHE_).^36^ The low onset potential and high photovoltage indicate a suppressed surface recombination and fast hole transport at the surface of the 3C/Ni(OH)_2_-aging photoanode. As compared in Table S1, the onset potential of 0.10 V_RHE_ achieved in the 3C/Ni(OH)_2_-aging photoanode is the lowest value among the mostly studied photoanodes including Si (*E*_onset_: 1.03–1.16 V_RHE_), α-Fe_2_O_3_ (*E*_onset_: 0.45–0.90 V_RHE_), BiVO_4_ (*E*_onset_: 0.40–0.60 V_RHE_), GaAs (*E*_onset_: 0.35–0.60 V_RHE_), and Ta_3_N_5_ (*E*_onset_: 0.38–0.75 V_RHE_). Note that the *E_onset_*values summarized in Table S1 were collected from both the single-junction and the buried-junction photoanodes, and the latter gives the smaller *E*_onset_ than the former owing to the larger built-in potential in the buried junction. For instance, it was shown that the Ta_3_N_5_ photoanode based on the buried junctions of *n-*type In:GaN/Ta_3_N_5_/*p*-type Mg:GaN exhibited a smaller onset potential (0.38 V_RHE_) than the single-junction Ta_3_N_5_ (0.75 V_RHE_) photoanode (Table S1).^37^ Impressively, the 3C/Ni(OH)_2_-aging as a single junction photoanode exhibits a much smaller onset potential than most of the buried-junction photoanodes (Table S1). This indicates that the 3C-SiC material offers substantial potential for achieving a high photovoltage.

Figure 2b shows the applied bias photon-to-current efficiency (ABPE) curves of 3C-SiC, 3C/Ni(OH)_2_, and 3C/Ni(OH)_2_-aging photoanodes. The 3C/Ni(OH)_2_-aging photoanode delivers the maximum ABPE of 0.66% at 0.62 V_RHE_, which is much higher than that of 3C/Ni(OH)_2_ (0.22% at 0.84 V_RHE_) and 3C-SiC (0.03% at 1.01 V_RHE_). Figure 2c compares the incident photon-to-current efficiency (IPCE) of 3C-SiC, 3C/Ni(OH)_2_, and 3C/Ni(OH)_2_-aging photoanodes. The 3C/Ni(OH)_2_-aging photoanode exhibits a high IPCE of ∼45% at wavelengths shorter than 450 nm, which is above its bandgap of 2.36 eV (Figure S1b). The photoresponse behavior of IPCE is consistent with the absorption spectrum of 3C-SiC (Figure S1a). At 450 nm, the IPCE values of 3C-SiC, 3C/Ni(OH)_2_, and 3C/Ni(OH)_2_-aging are 45%, 16%, and 3%, respectively. Clearly, the aged Ni(OH)_2_ cocatalyst dramatically enhances the photon conversion efficiency of the photoanode. To our knowledge, 45% of IPCE is the highest value ever reported for SiC-based photoanodes.^25,27,38−41^

### PEC Stability in Strongly Alkaline Solutions and In-Air Stability of 3C-SiC Photoanodes

Since practical water-splitting cells require continuous operation in either strongly alkaline or acidic electrolytes, the stability of the photoanodes was evaluated by measuring the photocurrent density–time (*J*–*t*) curves in 1.0 M NaOH (pH 13.6) solution at 1.0 V_RHE_ under illumination of AM1.5G, 100 mW/cm^2^ (Figure 2d). The 3C-SiC photoanode shows a fast degradation (52% loss of the initial photocurrent) within 400 s due to photocorrosion. The 3C/Ni(OH)_2_ shows a 31% degradation in photocurrent during the initial 15 min, but the photocurrent stabilizes afterward (Figure 2d,e). Impressively, the 3C/Ni(OH)_2_-aging photoanode achieves outstanding stability over 10 h of continuous *J*–*t* test in strongly alkaline electrolyte (1.0 M NaOH) and only exhibits a 7% loss of the initial photocurrent after the 10-h stability test (Figure 2d). As shown in Figure 2f, the *J*–*V* curves of the 3C/Ni(OH)_2_-aging photoanode before and after the 10-h stability test show the identical onset potential and a minor change (∼7%) in photocurrent. On the contrary, most of the studied photoanodes including Si, BiVO_4_, GaAs, GaP, and Ta_3_N_5_ commonly showed a dramatic degradation in photocurrent in strongly alkaline electrolyte (Table S1).

**Figure 3:**
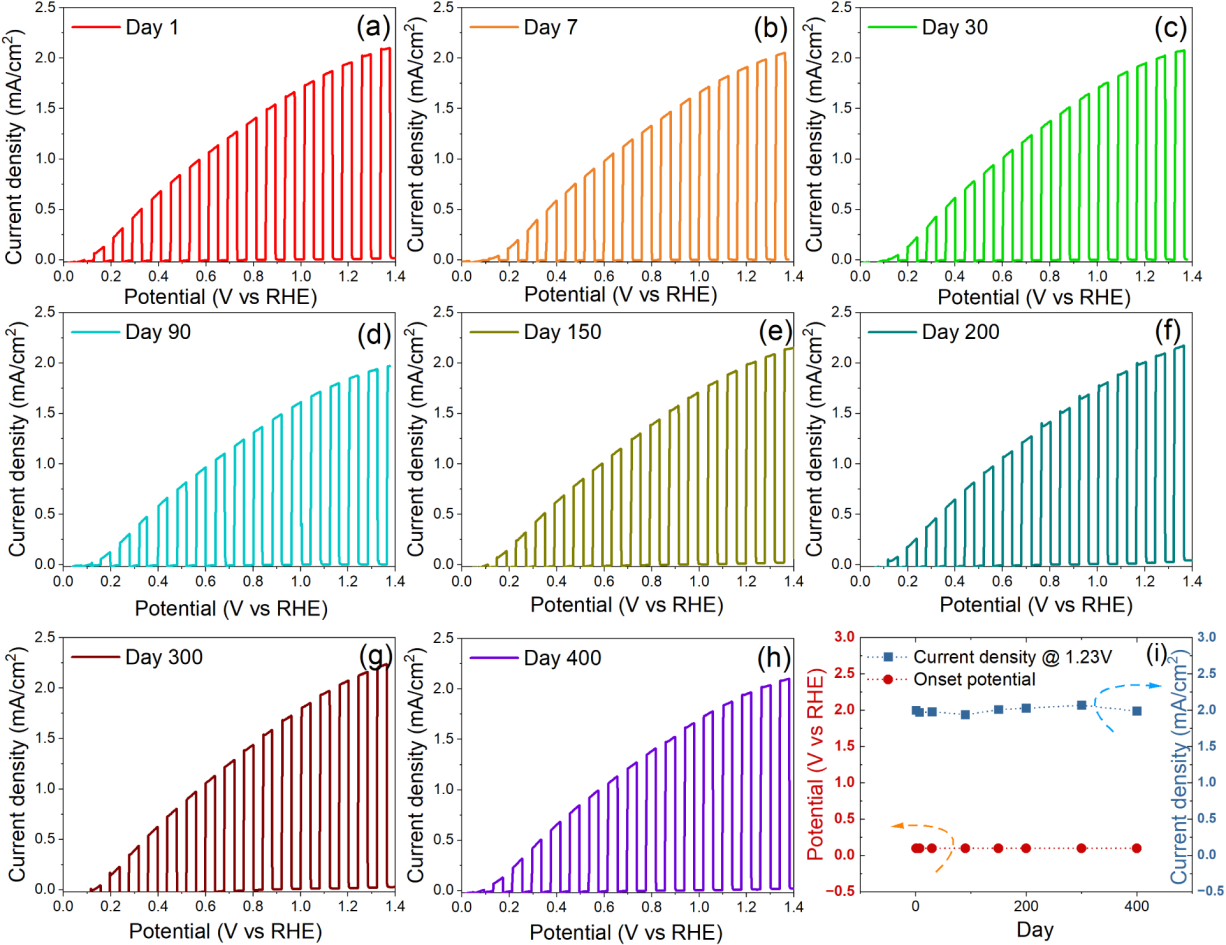
Current density–potential curves of 3C-SiC/Ni(OH)_2_-aging measured from day 1 to day 400 (a–h), and (i) the onset potential and photocurrent density at 1.23 V_RHE_ of the 3C-SiC/Ni(OH)_2_-aging photoanode over the course of 400 days.

In addition to the high stability in strongly alkaline electrolyte during PEC water splitting, the 3C/Ni(OH)_2_-aging photoanode also exhibits excellent long-term in-air stability over 400 days with identical PEC performance. Figure 3 compares the *J*–*V* curves of the freshly prepared and atmosphere-stored 3C/Ni(OH)_2_-aging photoanode over 400 days, where identical *J*–*V* curves can be observed. These results evidence that the 3C/Ni(OH)_2_-aging photoanode shows both outstanding PEC stability in corrosive alkaline electrolyte and long-term in-air stability.

### Photovoltage of 3C-SiC Photoanodes

**Figure 4:**
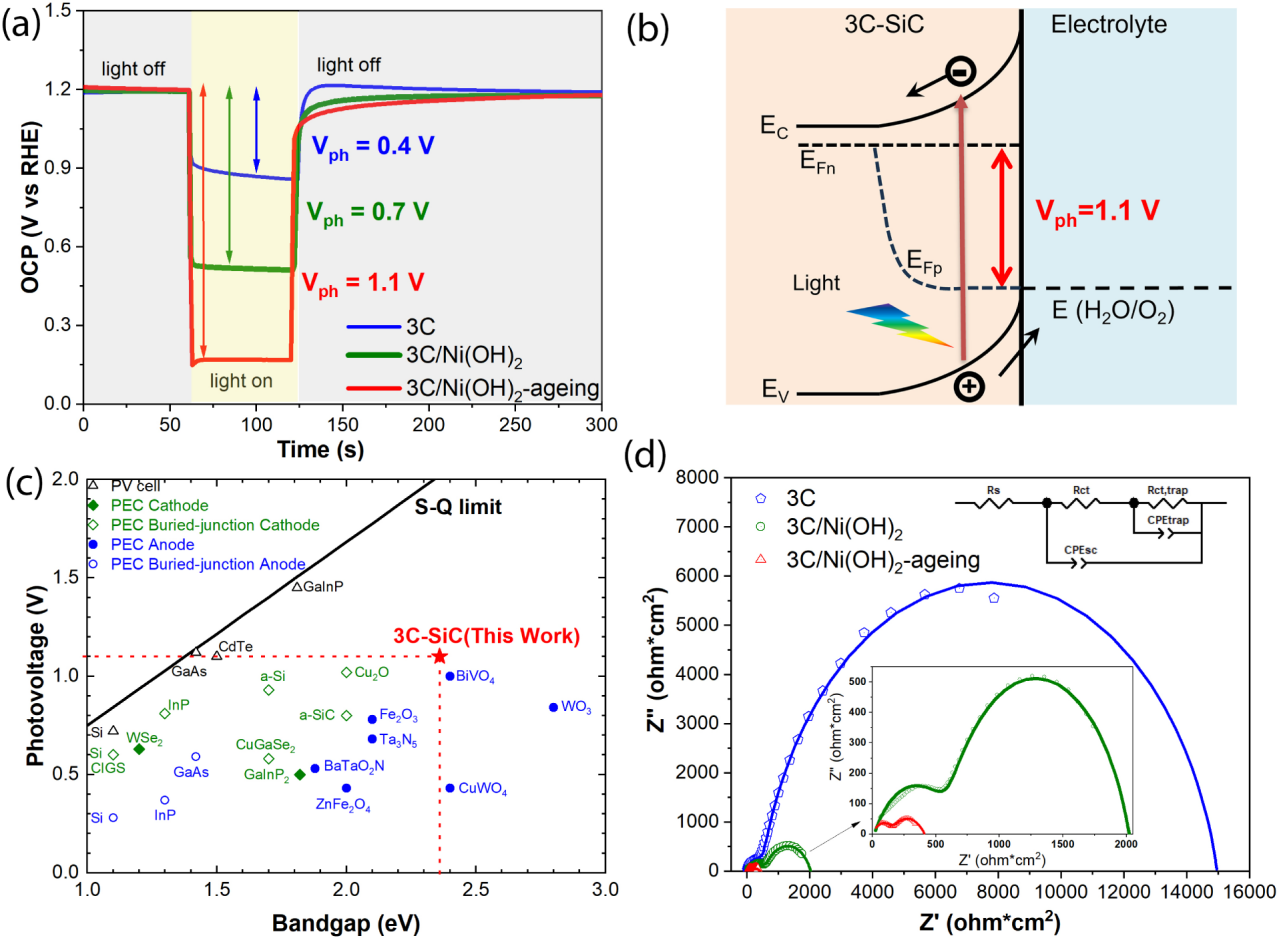
(a) Open-circuit potentials (OCPs) of 3C-SiC, 3C-SiC/Ni(OH)_2_, and 3C-SiC/Ni(OH)_2_-aging photoanodes measured under chopped AM1.5G 100 mW/cm^2^ illumination. (b) Schematic illustration of the photoanode/liquid band energetics under illumination, showing the generation of the photovoltage (V_ph_). E_C_: conduction band edge, E_V_: valence band edge, E_Fn_: electron quasi-Fermi level, E_Fp_: hole quasi-Fermi level. (c) Photovoltage benchmarks for PEC and photovoltaic (PV) materials as a function of the bandgap. Solid line represents the Shockley–Queisser (S–Q) photovoltage limit.^10^ Photovoltage data of PV cells, PEC cathodes, and anodes including their buried junctions are collected from reference.^11^ (d) Nyquist plots of the photoanodes at open-circuit conditions under AM1.5G 100 mW/cm^2^ illumination. The insets show the equivalent circuits to fit the impedance data, and the magnified Nyquist plots of 3C-SiC/Ni(OH)_2_ and 3C-SiC/Ni(OH)_2_-aging photoanodes.

To understand the improved PEC performance, the photovoltages of the 3C-SiC, 3C/Ni(OH)_2_, and 3C/Ni(OH)_2_-aging photoanodes were measured under open-circuit conditions (Figure 4a). In the dark, the equilibrium between the photoanode and the electrolyte results in the alignment of the Fermi level of the photoanode with the water oxidation potential. As a result, an identical open-circuit potential (OCP) of 1.23 V_RHE_ was observed in the dark for all three photoanodes (Figure 4a). Under illumination, the photogenerated electron–hole pairs are separated by the built-in electric field, and the photovoltage (V_ph_) is generated (Figure 4b). Thus, the OCPs of the photoanodes are negatively shifted under illumination. The photovoltage is determined by the difference between the measured OCPs in the dark and under illumination. Under AM1.5G 100 mW cm^–2^ illumination, the 3C/Ni(OH)_2_-aging photoanode achieves a high photovoltage of 1.10 V (Figure 4a), which is consistent with the V_ph_ of 1.13 V estimated from the onset potential shown in Figure 2a. In contrast, 3C-SiC and 3C/Ni(OH)_2_ show relatively low photovoltages of 0.40 and 0.70 V, respectively. Theoretically, the maximum V_ph_ of a semiconductor photoanode is determined by the difference between its flat band potential (E_FB_) and the water oxidation potential (1.23 V_RHE_).^36^ From the Mott–Schottky measurements, we obtain an identical E_FB_ of −0.26 V_RHE_ for the 3C-SiC, 3C/Ni(OH)_2_, and 3C/Ni(OH)_2_-aging photoanodes (Figure S5). Therefore, a maximum V_ph_ of 1.49 V is expected for the 3C-SiC photoanode. However, the measured V_ph_ is usually lower than the maximum value due to bulk and surface recombination. For the 3C-SiC, 3C/Ni(OH)_2_, and 3C/Ni(OH)_2_-aging photoanodes, the charge recombination in the 3C-SiC bulk is expected to be equivalent since the 3C-SiC was grown under the same conditions. Therefore, the dramatic increase in the photovoltage at the 3C/Ni(OH)_2_-aging should be due to the suppression of the surface recombination of the photoanode. This result agrees very well with the enhanced photocurrent and IPCE of the 3C/Ni(OH)_2_-aging photoanode.

Figure 4c summarizes the best-reported photovoltages for commonly studied photovoltaic (PV) and PEC materials as a function of the bandgap.^11^ Compared to the PV materials with photovoltages approaching their Shockley–Queisser photovoltage limits,^10^ the reported PEC photoanodes and cathodes including those with buried junctions showed much lower photovoltages (less than 1 V) regardless of their bandgaps. Impressively, the photovoltage of 1.10 V achieved in the 3C/Ni(OH)_2_-aging photoanode is the highest value among the reported photoelectrodes (Figure 4c).

To get insight into the enhanced photovoltage, the carrier transport properties across the photoanode/electrolyte interface was studied by measuring the electrochemical impedance spectroscopy (EIS) at open-circuit conditions under AM1.5G 100 mW/cm^2^ illumination. Figure 4d compares the Nyquist plots of the 3C-SiC, 3C/Ni(OH)_2_, and 3C/Ni(OH)_2_-aging photoanodes. The EIS data are fitted using the equivalent circuits shown in the inset in Figure 4d and the fitted values are summarized in Table S2. We find that the 3C/Ni(OH)_2_-aging photoanode exhibits a much smaller charge-transfer resistance across the photoanode/electrolyte interface (R_ct,trap_ = 247 Ω cm^2^) than 3C-SiC (R_ct,trap_ = 14350 Ω cm^2^) and 3C/Ni(OH)_2_ (R_ct,trap_ = 1370 Ω cm^2^). This result indicates that aged Ni(OH)_2_ catalyst reduces the charge recombination and promotes the hole transfer efficiency at the photoanode/electrolyte interface, thus significantly enhancing the photovoltage of the 3C/Ni(OH)_2_-aging photoanode.

### Understanding the Aging Effect of Ni(OH)_2_ on the Improvement of the PEC Performance

**Figure 5:**
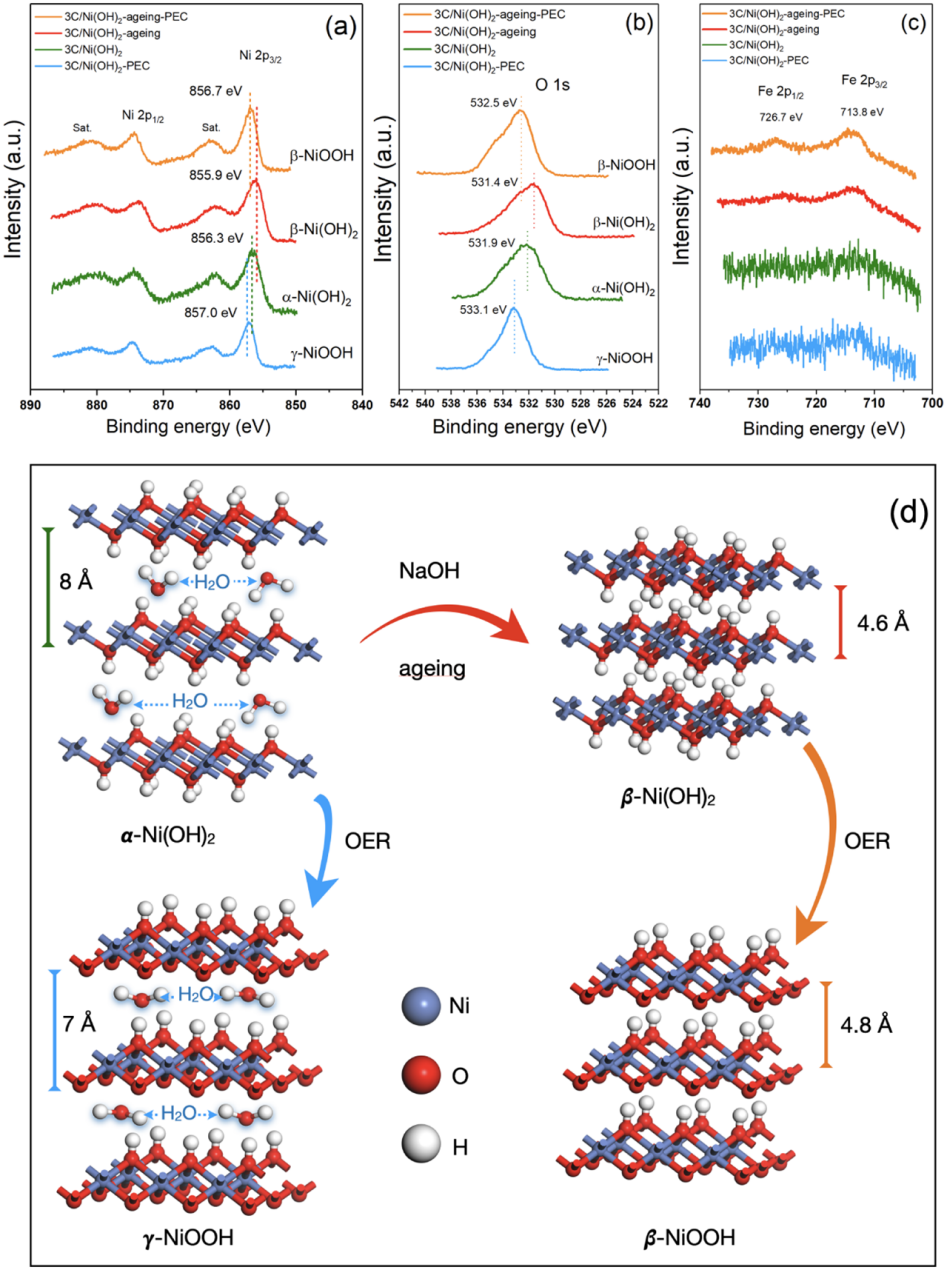
XPS spectra of (a) Ni 2p, (b) O 1s, and (c) Fe 2p of 3C-SiC/Ni(OH)_2_ and 3C-SiC/Ni(OH)_2_-aging before and after PEC stability tests. The two samples after PEC stability tests are denoted as 3C-SiC/Ni(OH)_2_-PEC and 3C-SiC/Ni(OH)_2_-aging-PEC, respectively. (d) Schematic illustration of phase transitions of Ni(OH)_2_ induced by the aging process and PEC or OER tests.

To better understand the aging effect of Ni(OH)_2_ on the improvement of the overall PEC performance, we comparatively investigated the morphology and chemical states of the 3C/Ni(OH)_2_ and 3C/Ni(OH)_2_-aging photoanodes before and after PEC stability tests (Figure 5). Hereafter, these two photoanodes after PEC stability tests are donated as 3C/Ni(OH)-PEC and 3C/Ni(OH)-aging-PEC, respectively. The surface morphology of 3C/Ni(OH)-PEC is completely changed after PEC tests, showing more coherent networks compared to the nanoparticle layer of the as-prepared 3C/Ni(OH)_2_ (Figure S6a,c). After PEC stability tests, the 3C/Ni(OH)_2_-aging-PEC exhibited distinct nanosheets compared with large nanoparticle grains of 3C/Ni(OH)_2_-aging (Figure S6b,d). The EDX (Si, C, O and Ni maps) results of the 3C/Ni(OH)_2_ and 3C/Ni(OH)_2_-aging showed negligible difference before and after the PEC stability tests (Figures 1e,f and S7).

It is known that Ni(OH)_2_ has two polymorphs, namely, α-Ni(OH)_2_ and β-Ni(OH)_2_. The β-phase is isostructural with brucite, while α-Ni(OH)_2_ consists of β-Ni(OH)_2_ layers intercalated by water molecules.^42^ As confirmed by Raman measurements below, the as-prepared Ni(OH)_2_ is the α-phase and the aged Ni(OH)_2_ is the β-phase, while after PEC stability tests, both of them are oxidized to NiOOH (α-Ni(OH)_2_ → γ-NiOOH and β-Ni(OH)_2_ → β-NiOOH),^42^ which are illustrated in Figure 5d. These phase transitions explain the morphological changes in photoanodes induced by the aging effect and PEC tests.

Figures 5a–c and S8 compare the XPS spectra of Ni 2p, O 1s, and Fe 2p of the 3C-SiC/Ni(OH)_2_ and 3C-SiC/Ni(OH)_2_-aging before and after PEC stability tests. As shown in Figure 5a, the Ni 2p_3/2_ peak of the as-prepared 3C/Ni(OH)_2_ is located at 856.3 eV, indicating the presence of Ni^2+^. After aging, the Ni 2p_3/2_ peak in 3C/Ni(OH)_2_-aging is shifted to a low energy position of 855.9 eV. This low-energy shift is consistent with the phase transition from α- to β-Ni(OH)_2_.^43−45^ After the PEC tests, the Ni 2p_3/2_ peaks of 3C/Ni(OH)_2_-PEC and 3C/Ni(OH)_2_-aging-PEC are shifted to higher energy positions of 857.0 and 856.7 eV, respectively, indicating the existence of Ni^3+^ (formation of NiOOH). Moreover, the highest energy state of 857.0 eV in 3C/Ni(OH)_2_-PEC suggests the formation of γ-NiOOH and the 0.3 eV energy downshift suggests the formation of β-NiOOH in 3C/Ni(OH)_2_-aging-PEC. These results agree with the reported energy positions and shifts of Ni 2p spectra induced by the phase transitions of Ni(OH)_2_.^43,46^

Consistent with the shifts of Ni 2p peaks, the energy shifts of the O 1s spectra also support the phase transitions of Ni(OH)_2_ (Figure 5b). The O 1s peak of the as-prepared Ni(OH)_2_ is located at 531.9 eV, which is shifted to 531.4 eV for aged Ni(OH)_2_. These peaks are corresponding to hydroxyl and the low-energy shift agrees very well with the phase transition from α-Ni(OH)_2_ to β-Ni(OH)_2_.^43,44^ After the PEC tests, the 3C/Ni(OH)_2_-PEC and 3C/Ni(OH)_2_-aging-PEC show peaks at 533.1 and 532.5 eV, respectively, which are attributed to the presence of -OOH and consistent with previously reported results in γ-NiOOH and β-NiOOH.^46^ It is also worth noting that very weak peaks of Fe 2p can be observed in the aged samples, whereas not observed in the unaged 3C/Ni(OH)_2_ (Figure 5c). It has been suggested that Fe can be incorporated into Ni(OH)_2_ from the impurities in NaOH during the aging process and the incorporation of Fe can enhance the OER performance of Ni(OH)_2_.^47,48^

### Electrocatalytic Oxygen Evolution Reaction (OER) of Ni(OH)_2_ Catalysts

**Figure 6:**
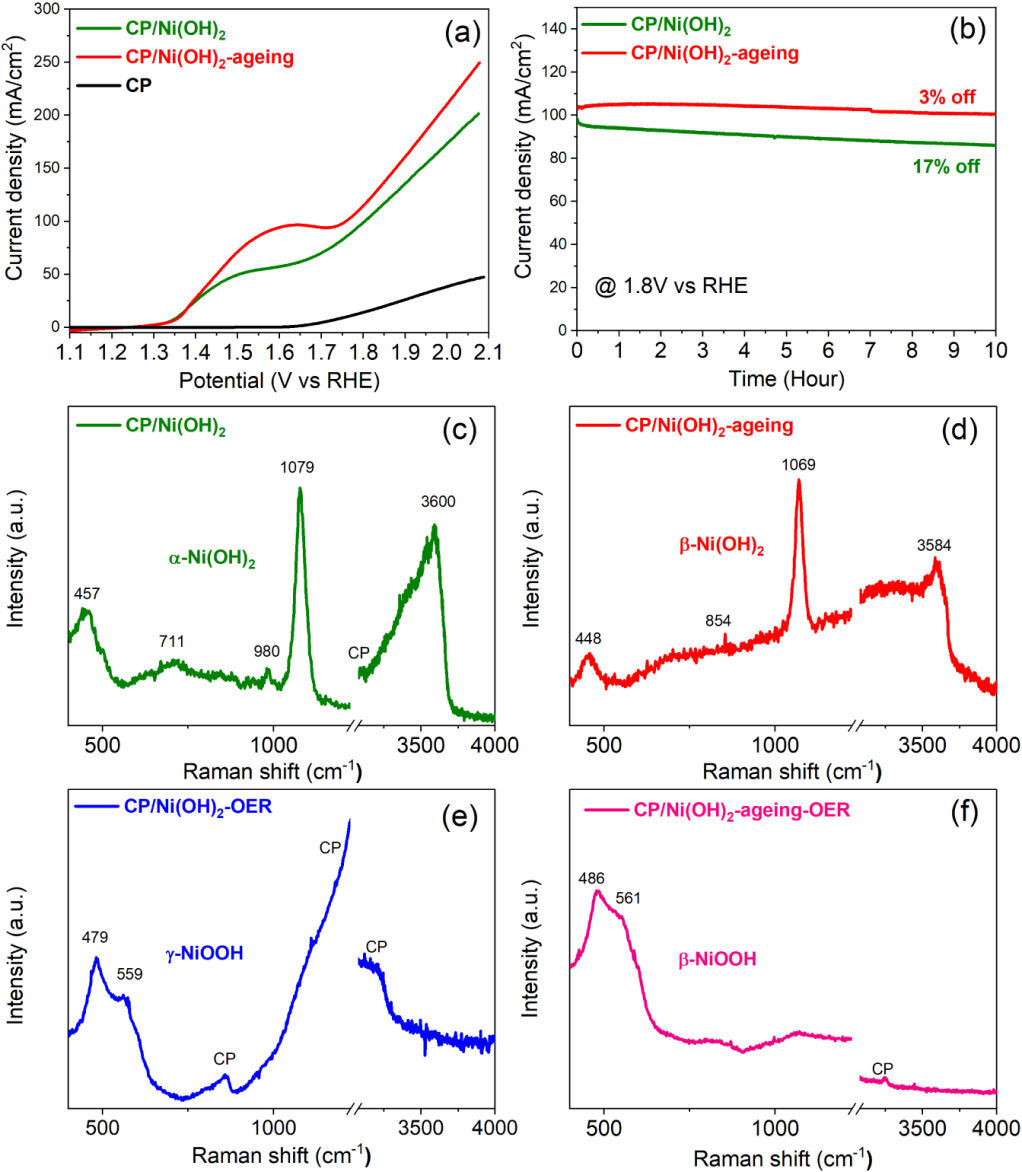
(a) LSV curves of the Ni(OH)_2_ grown on carbon paper (CP) before and after aging in 1.0 M NaOH solution for 40 h. The LSV curve of the bare CP is shown as a comparison. (b) Chronoamperometry stability tests of CP/Ni(OH)_2_ and CP/Ni(OH)_2_-aging at the potential of 1.8 V_RHE_ for 10 h. Raman spectra of (c) CP/Ni(OH)_2_, (d) CP/Ni(OH)_2_-aging, (e) CP/Ni(OH)_2_ after OER tests, and (f) CP/Ni(OH)_2_-aging after OER tests. The Raman spectrum of the bare carbon paper is shown in Figure S11.

To get further insights into the OER activity of the Ni(OH)_2_ catalysts, we adopt the same hydrothermal method to synthesize Ni(OH)_2_ on the chemically inert carbon paper (CP) fibers and compare the OER performance before and after the aging process. The as-prepared Ni(OH)_2_ on CP is denoted as CP/Ni(OH)_2_, and the CP/Ni(OH)_2_ after aging in 1 M NaOH for 40 h without applying any bias is denoted as CP/Ni(OH)_2_-aging. Linear sweep voltammetry (LSV) measurements show that CP/Ni(OH)_2_-aging exhibits better OER activity than CP/Ni(OH)_2_ (Figure 6a). The stability of the electrodes was evaluated by measuring chronoamperometry at 1.8 V_RHE_ in 1.0 M NaOH (pH 13.6) solution for 10 h. As shown in Figure 6b, the CP/Ni(OH)_2_-aging electrode shows a significantly enhanced stability with a minor (∼3%) loss of initial current density after the continuous *J*–*t* test for 10 h, which is much better than that of the unaged CP/Ni(OH)_2_ (17% loss of initial current). Clearly, the improved OER activity and stability of the aged Ni(OH)_2_ both agree very well with the enhanced PEC performance of the 3C/Ni(OH)_2_-aging photoanode (Figure 2). These results indicate that the facile aging process is an effective approach to achieve highly active and stable OER catalysts for both electrocatalytic and photoelectrochemical oxygen evolution in strongly alkaline solutions.

As seen in Figure 6a, the as-prepared CP/Ni(OH)_2_ shows an oxidation peak at ∼1.5 V_RHE_, which is attributed to the oxidation of Ni^2+^ to Ni^3+^ owing to the phase transition of α-Ni(OH)_2_ to γ-NiOOH during the OER process.^43,49,50^ For the aged sample, the Ni^2+^ to Ni^3+^ oxidation peak is shifted to a higher potential (∼1.6 V_RHE_), which is attributed to the phase transition from β-Ni(OH)_2_ to β-NiOOH.^50^ This result indicates that the crystalline structures of β-Ni(OH)_2_ and β-NiOOH are more stable than those of α-Ni(OH)_2_ and γ-NiOOH because the latter two structures contain intercalated water molecules (Figure 5d).^42^

The phase transitions of Ni(OH)_2_ induced by the aging process and OER tests are further confirmed by the Raman spectroscopy. The Raman spectrum of the as-prepared CP/Ni(OH)_2_ show peaks at 457, 711, 980, 1079, and 3600 cm^–1^, which are characteristic of α-Ni(OH)_2_ (Figure 6c).^42^ The 457 cm^–1^ peak is ascribed to the lattice Ni–OH stretching mode, and the strong and broad band at 3600 cm^–1^ is assigned to the O–H stretching mode from intercalated H_2_O of α-Ni(OH)_2_.^42^ After aging, the CP/Ni(OH)_2_-aging shows peaks of 448, 854, 1069, and 3584 cm^–1^, which are typical modes of β-Ni(OH)_2_ (Figure 6d). Particularly, the shift in the major lattice Ni–OH stretching mode from 457 to 448 cm^–1^, the absence of the O–H stretching mode from intercalated H_2_O at 3600 cm^–1^, and the appearance of the O–H stretching mode at 3584 cm^–1^ are characteristics of the phase transition from α- to β-Ni(OH)_2_.^51−54^

For the as-prepared Ni(OH)_2_ after OER tests, it exhibits two peaks at 479 and 559 cm^–1^, indicating the formation of γ-NiOOH (Figure 6e).^55^ The absence of other peaks such as the second-order lattice mode at 1079 cm^–1^ and the O–H stretching mode at 3600 cm^–1^ further evidence the phase transition from α-Ni(OH)_2_ to γ-NiOOH.^55^ For the aged Ni(OH)_2_ after OER tests, it shows two peaks at 486 and 561 cm^–1^ (Figure 6f), which are the characteristic peaks of β-NiOOH.^54^ Moreover, the aged Ni(OH)_2_ after OER tests clearly shows nanosheets formed on carbon paper fibers (Figure S9), consistent with the morphological changes in the 3C/Ni(OH)_2_-aging after PEC tests (Figure S6). EDX maps confirm the presence of the Ni and the O elements of the deposited layer on carbon paper fibers (Figure S10). These results further support the formation of β-NiOOH on the aged samples after PEC or OER tests.

The XPS and Raman results clearly reveal that the as-prepared α-Ni(OH)_2_ is oxidized to γ-NiOOH after the OER reaction while the aging process converts α-Ni(OH)_2_ to β-Ni(OH)_2_, accompanied by the incorporation of Fe into β-Ni(OH)_2_, which is further oxidized to β-NiOOH after the OER reaction. Compared to the loosely stacked α-Ni(OH)_2_ and γ-NiOOH that exhibit a larger interlayer distance of 7–8 Å due to the intercalated H_2_O molecules, the β-Ni(OH)_2_ and β-NiOOH exhibit more stable and compactly stacked structures, showing decreased interlayer distances of 4.6–4.8 Å due to the removal of intercalated H_2_O molecules (Figure 5d). Therefore, the aging process is critical for the formation of more stable and highly active Fe-doped β-NiOOH,^42,47,48^ which accounts for the enhanced OER activity and stability for PEC water oxidation.

## Conclusions

In summary, we have demonstrated a simple and effective approach to fabricate high-photovoltage and stable photoanodes for solar water splitting in strongly alkaline solutions. With the deposition of Ni(OH)_2_ on 3C-SiC, followed by aging in 1.0 M NaOH at room temperature for 40 h without applying electrochemical bias, the resulting photoanode achieves a record-high photovoltage of 1.10 V and an ultralow onset potential of 0.10 V_RHE_ for PEC water oxidation, distinctly outperforming the pristine 3C-SiC and its counterpart without aging. Moreover, the aged 3C-SiC/Ni(OH)_2_ photoanode shows outstanding operational stability in strongly alkaline electrolyte (pH 13.6) and long-term in-air stability without degradation of its PEC performance for more than 400 days. The IPCE and EIS results reveal that aged Ni(OH)_2_ catalyst dramatically promotes the hole transport at the photoanode/electrolyte interface, thus significantly enhancing the overall PEC water oxidation performance. Furthermore, we synthesized Ni(OH)_2_ on the chemically inert carbon paper (CP) fibers following the same aging process. Consistent with the improved PEC performance of the 3C-SiC/Ni(OH)_2_-aging photoanode, the aged CP/Ni(OH)_2_ catalyst also showed much better OER activity and stability than its counterpart without aging. The XPS and Raman results evidence that the as-prepared α-Ni(OH)_2_ is converted into γ-NiOOH after OER tests, while the aging process converts α-Ni(OH)_2_ into β-Ni(OH)_2_ accompanied by Fe incorporation from the impurities of NaOH, which is further oxidized to Fe-doped β-NiOOH after OER tests. Compared to the unstable α-Ni(OH)_2_ and γ-NiOOH, the Fe-doped β-NiOOH induced by the aging process and subsequent OER reactions is more stable and highly active, thus enhancing the overall PEC performance and stability for solar water splitting. This work demonstrates a facile aging process to fabricate high photovoltage and stable photoanodes for PEC water splitting in strongly alkaline solutions.

## Experimental Section

### Material Synthesis

High crystalline quality 3C-SiC (∼300 um thick) layers were grown on 1.2 degree off-axis 6H-SiC(0001) substrates by the sublimation technique.^29−31^ The as-prepared 3C-SiC samples were chemically cleaned with ethanol, acetone, H_2_O:H_2_O_2_:NH_3_ (5:1:1), H_2_O:H_2_O_2_:HCl (6:1:1), and 5% hydrofluoric acid solution in order. To form Ohmic contacts, 60 nm Ni and 300 nm Au were deposited on the backside of the 3C-SiC/6H-SiC, followed by annealing in Ar at 900 °C for 10 min. Then, the 3C-SiC photoanodes were sealed with epoxy so that only the surface is exposed to solution for light illumination in PEC measurements.

### Preparation of 3C-SiC/Ni(OH)_2_ Photoanodes

A layer of Ni(OH)_2_ was deposited on 3C-SiC by a previously reported hydrothermal method.^32^ A 25 mL homogeneous solution containing 100 mM NiCl_2_ and 45 mM urea was first prepared. Along with 3C-SiC photoanodes, the solution was transferred into a 25 mL autoclave and sealed. The autoclave was heated up to 100 °C for 2 h. After natural cooling to room temperature, the 3C-SiC/Ni(OH)_2_ photoanodes were rinsed with distilled water and dried in air.

### Preparation of 3C-SiC/Ni(OH)_2_-Aging Photoanodes

Aging of 3C-SiC/Ni(OH)_2_ photoanodes was performed by immersing the photoanodes in 1.0 M NaOH solution for 40 h at room temperature without applying electrochemical potential. We found that the 3C-SiC/Ni(OH)_2_ photoanode with aging for 40 h gives the best PEC performance. Afterward, the 3C-SiC/Ni(OH)_2_-aging photoanodes were rinsed with distilled water and dried in air.

### Preparation of CP/Ni(OH)_2_ and CP/Ni(OH)_2_-Aging Electrodes

Prior to the synthesis, carbon paper (CP) was cleaned with acetone and HCl solution (8%), respectively. CP/Ni(OH)_2_ electrodes were fabricated using the same hydrothermal method as that of 3C-SiC/Ni(OH)_2_ by replacing the 3C-SiC photoanode with carbon papers (2 × 1 cm^2^). The CP/Ni(OH)_2_-aging electrodes were prepared by immersing CP/Ni(OH)_2_ electrodes in 1.0 M NaOH solution for 40 h at room temperature without applying electrochemical potential.

### Characterizations

Scanning electron microscopy (SEM) and energy-dispersive X-ray spectroscopy (EDX) measurements were done by a LEO 1550 Gemini instrument. X-ray photoelectron spectroscopy (XPS) was performed on the samples without Ar+ etching using a Scienta-200 hemispherical analyzer with monochromatized Al Kα line (1486.6 eV). The photoelectron spectroscopy measurements were performed in vacuum with background pressure lower than 1 × 10^–9^mbar. Raman measurements were performed using a homemade micro-Raman setup with 532 nm laser exication and a monochromator (Jobin Yvon HR460) coupled to a CCD camera.

### Photoelectrochemical Measurements

PEC measurements were performed in a three-electrode cell by an electrochemical station (Princeton Applied Research, VersaSTAT 3) in 1.0 M NaOH solution (pH = 13.6) under AM1.5G 100 mW/cm^2^ illumination from the solar simulator (LOT-Quantum Design GmbH). The solar simulator was calibrated by a standard Si photovoltaic cell prior to the measurement. The 1.0 M NaOH solution was deoxygenated by bubbling with Ar gas (99.999%) for over 30 min before measurement. The 3C-SiC–based photoanodes, Ag/AgCl (saturated KCl), and 1 × 1 cm^2^ Pt plate were used as the working electrode, the reference electrode, and the counter electrode. The current density versus potential (*J*–*V*) measurements were performed at a scan rate of 20 mV/s. The measured potential (*V*_Ag/AgCl_) with respect to the reference electrode were converted to the potential versus reversible hydrogen electrode (*V*_RHE_) using the following equation:  The applied bias photon-to-current efficiency (ABPE) was calculated using the equation:  , where *J* is the photocurrent density at the potential *V*, and P_AM1.5G_ is the light density of simulated sunlight (AM1.5G, 100 mW/cm^2^). The incident photon-to-current efficiency (IPCE) was measured in 1.0 M NaOH solution at 1.0 *V*_RHE_ under monochromatic light illumination produced from the solar simulator using a series of 10 nm bandpass filters (Thorlabs). The output of the monochromatic light was measured by a photodiode detector (Thorlabs). The open-circuit potential (OCP) measurements were performed in 1.0 M NaOH with the continuous bubbling of O_2_ under chopped illumination of AM1.5G, 100 mW/cm^2^. The electrochemical impedance spectroscopy (EIS) was conducted in the frequency range of 1–10^5^ Hz at the open-circuit conditions under AM1.5G, 100 mW/cm^2^ illumination.

### Electrochemical Measurements

The electrochemical oxygen evolution reaction (OER) measurements were performed in 1.0 M NaOH solution (pH = 13.6), where the CP/Ni(OH)_2_-based electrode was used as the working electrode, Hg/HgO was used as the reference electrode, and Pt was used as the counter electrode. The potential versus RHE is calculated using the following equation: . The linear sweep voltammetry (LSV) measurements of CP/Ni(OH)_2_-based electrodes were recorded at a scan rate of 10 mV/s.

## Supporting Information Available

Experimental details, structural and compositional characterization, (photo)electrochemical properties, XPS and Raman results (PDF)
