# Supplementary material for: Aging Ni(OH)2 on 3C-SiC Photoanodes to Achieve a High Photovoltage of 1.1 V and Enhanced Stability for Solar Water Splitting in Strongly Alkaline Solutions
Source: ACS Appl Mater Interfaces. 2024 Sep 17;16(38):50926–36. doi: 10.1021/acsami.4c11809 (PMC11440461; doi:10.1021/acsami.4c11809)
Supplement: Supplementary file 1 — am4c11809_si_001.pdf [file am4c11809_si_001.pdf]

Supporting Information

Ageing $Ni(OH)_2$ on 3C-SiC photoanodes to achieve a high photovoltage of 1.1 V and enhanced stability for solar water splitting in strongly alkaline solutions

Yuanju Qu,[a] Valdas Jokubavicius,[a] Duc Quang Hoang,[a] Xianjie Liu,[b] Mats Fahlman,[b] Ivan G. Ivanov,[a] Rositsa Yakimova,[a] and Jianwu Sun*[a]

[a] *Department of Physics, Chemistry and Biology (IFM), Linköping University, SE-58183, Linköping, Sweden*

[b] *Laboratory of Organic Electronics, Department of Science and Technology, Linköping University, Norrköping, 60174, Sweden*

* Corresponding Author: Jianwu Sun, jianwu.sun@liu.se

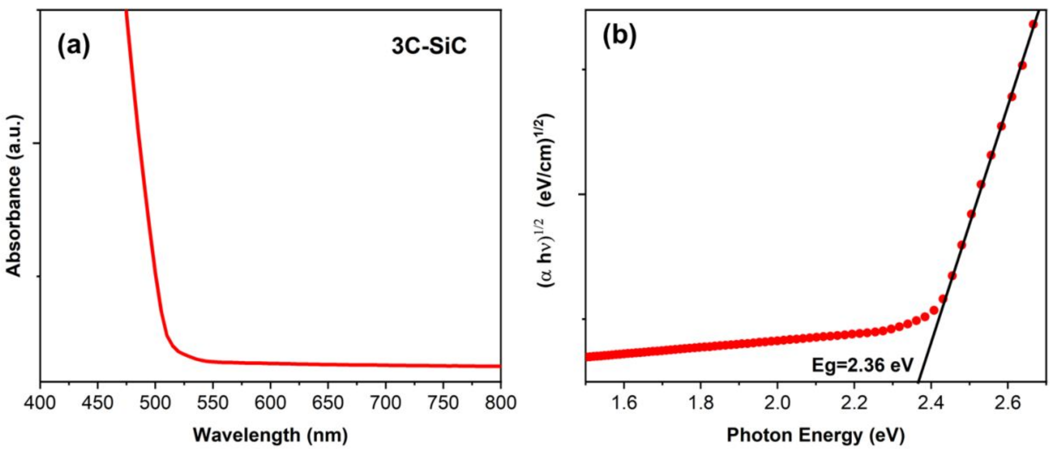

Fig. S1 Absorption spectrum (a) and Tauc plot (b) of the as-grown 3C-SiC.

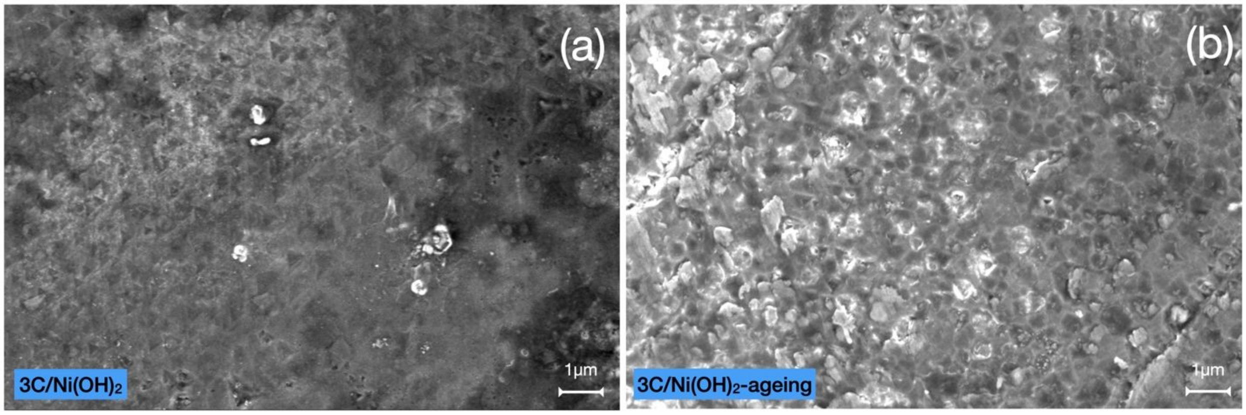

Fig. S2 SEM images of (a) 3C-SiC/$Ni(OH)_2$ and (b) 3C-SiC/$Ni(OH)_2$-ageing with a scale bar of 1 μm.

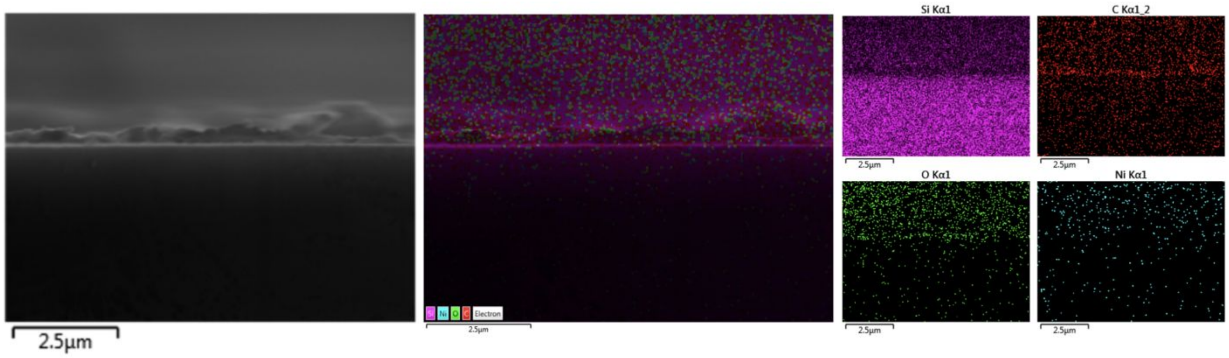

Fig. S3 Cross-sectional EDX mappings of Si, C, O, and Ni elements of the as-prepared 3C-SiC/$Ni(OH)_2$.

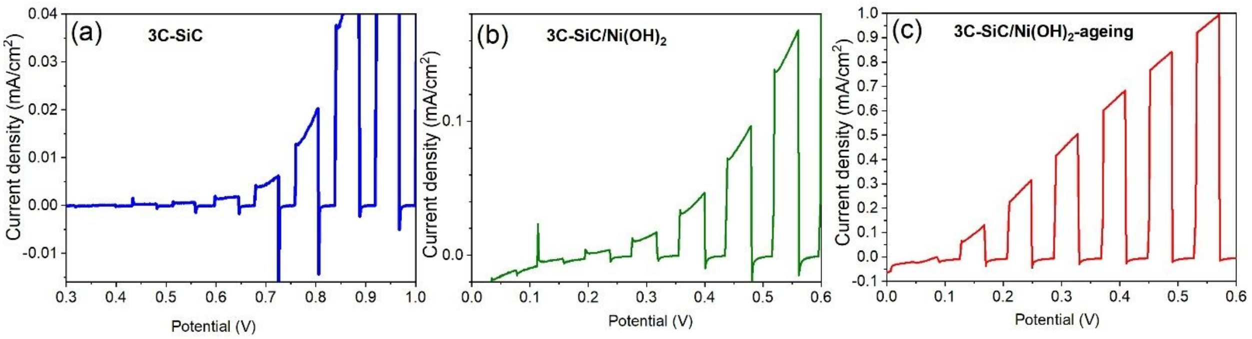

Fig. S4 Photocurrent density–potential (J–V) curves of 3C-SiC (a), 3C-SiC/Ni(OH)$_2$ (b) and 3C-SiC/Ni(OH)$_2$-ageing (c) photoanodes measured in 1.0 M NaOH electrolyte under chopped AM1.5G 100 mW/cm^2 illumination, at a scan rate of 20 mV/s. The 3C-SiC and 3C-SiC/Ni(OH)$_2$ photoanodes show the transient photocurrent when switching light on/off. In contrast, the SiC/Ni(OH)$_2$-ageing photoanode shows negligible transient photocurrent.

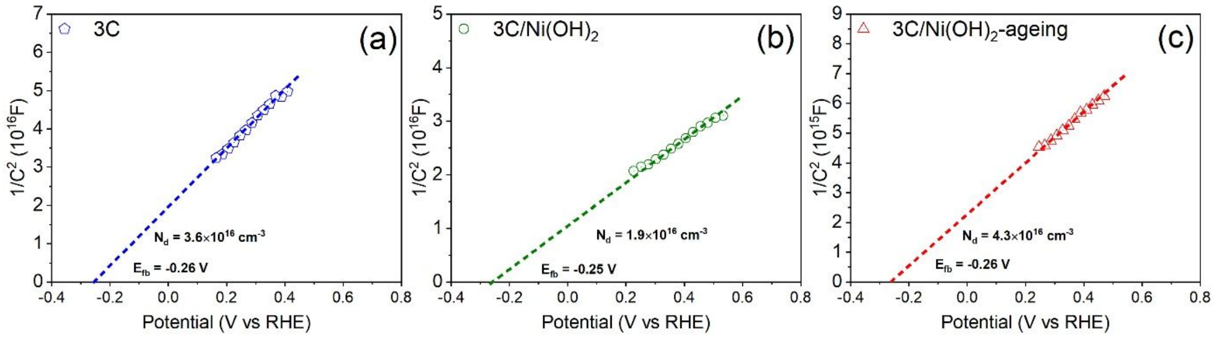

Fig. S5 Mott-Schottky plots of (a) 3C-SiC, (b) 3C-SiC/Ni(OH)$_2$ and (c) 3C-SiC/Ni(OH)$_2$-ageing photoanodes. The flat-band potentials were obtained from the intercepts of the extrapolated lines.

The doping concentration N_d was derived from the slope by the equation: $N_d = \frac{2}{\varepsilon_0 \varepsilon_r e A^2}\left[\frac{d(\frac{1}{C^2})}{dV}\right]^{-1}$, where ε_0 is vacuum permittivity, where ε_r is the dielectric constant of 3C-SiC, A is the surface area, e is the elementary charge.

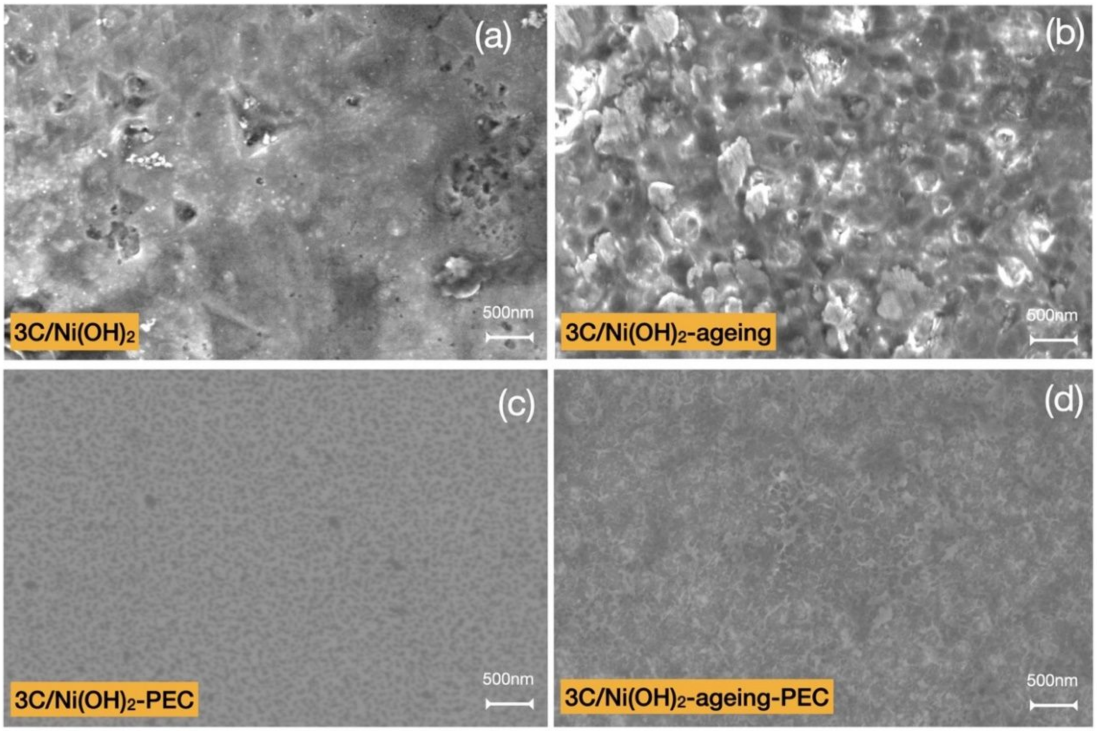

Fig. S6 SEM images of (a) the as-prepared 3C-SiC/Ni$(OH)_2$ and (b) 3C-SiC/Ni$(OH)_2$-ageing that was aged in 1.0 M NaOH solution for 40 hours, (c) 3C-SiC/Ni$(OH)_2$ after PEC stability tests, and (d) 3C-SiC/Ni$(OH)_2$-ageing after PEC stability tests.

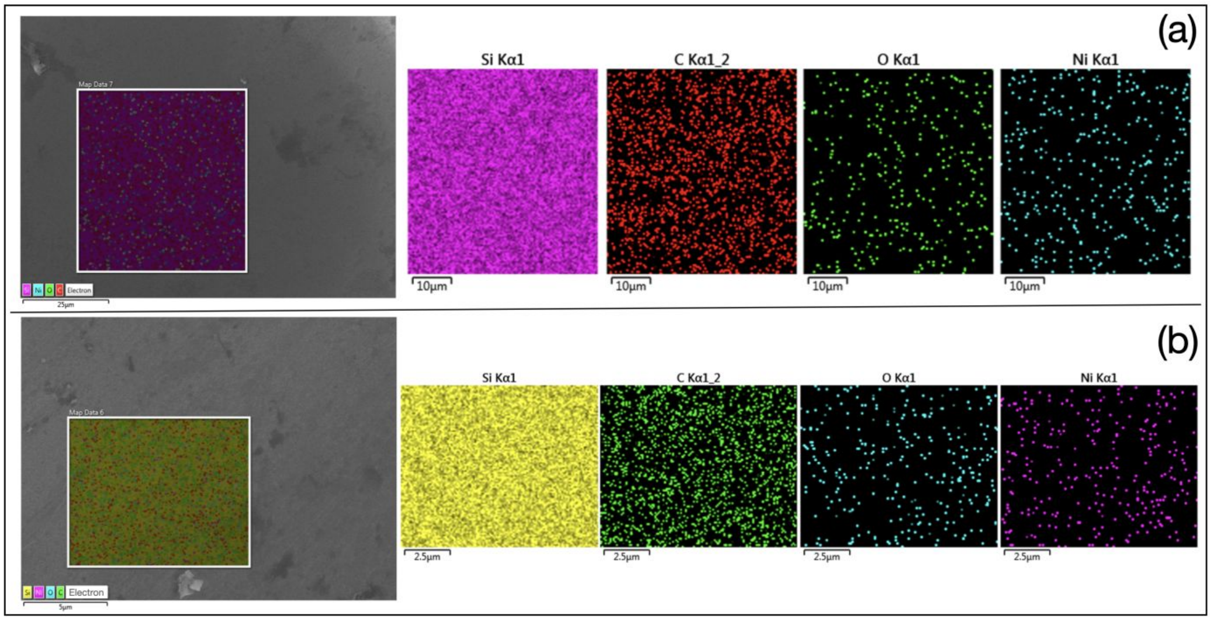

Fig. S7 EDX of (a) 3C-SiC/Ni$(OH)_2$ after PEC stability tests and (b) 3C-SiC/Ni$(OH)_2$-ageing after PEC stability tests.

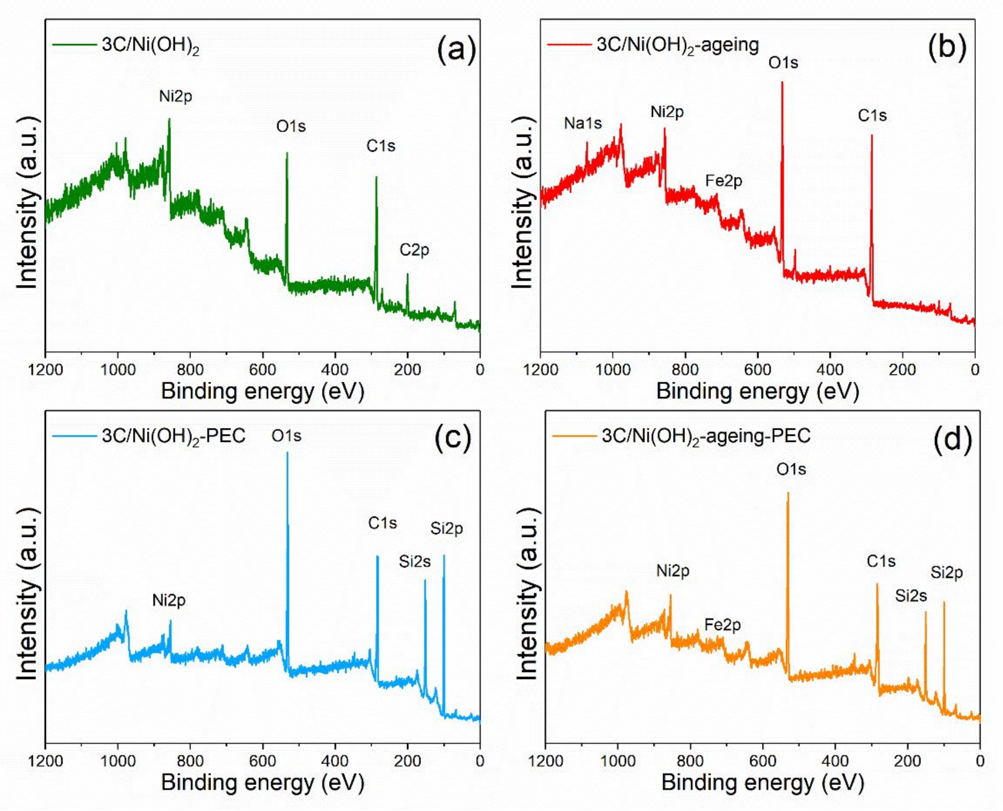

Fig. S8 XPS survey spectra of (a) the as-prepared 3C-SiC/$Ni(OH)_2$, (b) 3C-SiC/$Ni(OH)_2$-ageing that was aged in 1.0 M NaOH solution for 40 hours, (c) 3C-SiC/$Ni(OH)_2$ after PEC stability tests, and (d) 3C-SiC/$Ni(OH)_2$-ageing after PEC stability tests.

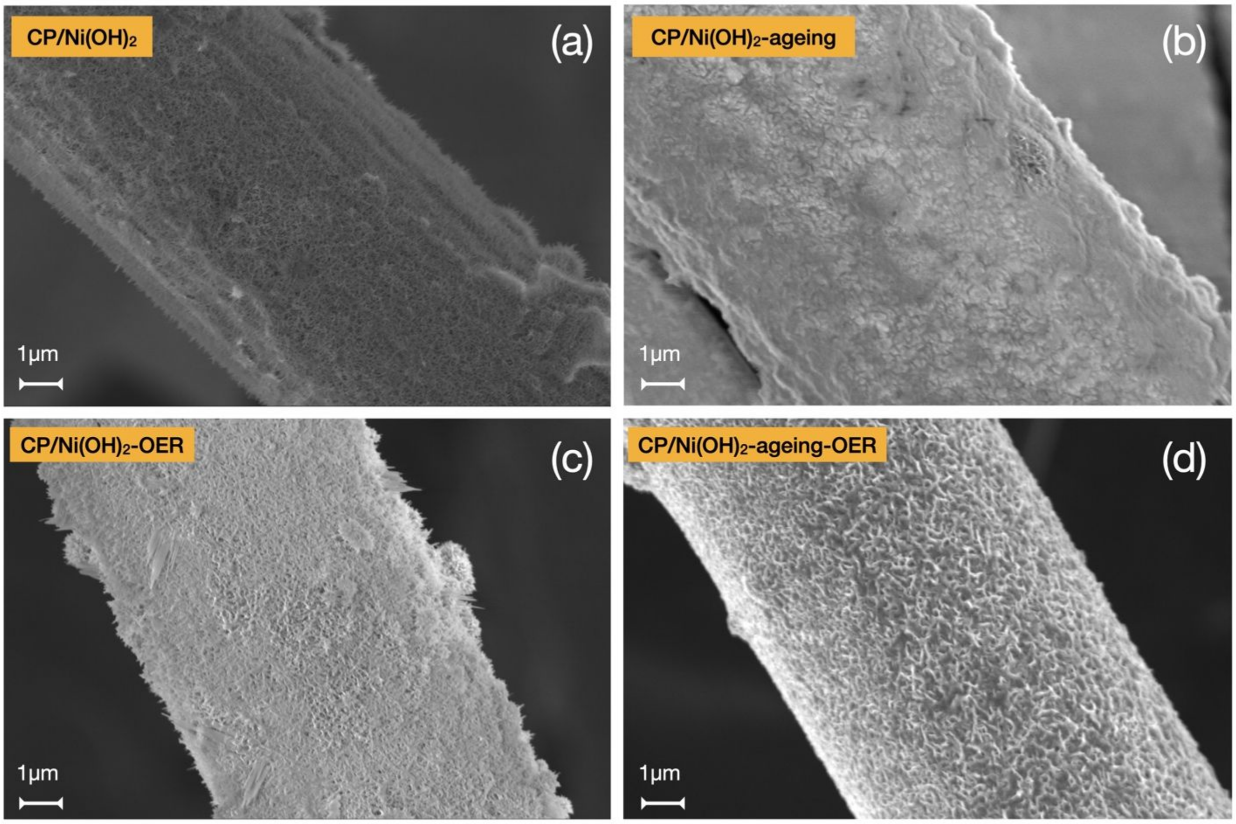

Fig. S9 SEM images of (a) the as-prepared $Ni(OH)_2$ on carbon paper (CP) fibers, (b) CP/$Ni(OH)_2$-ageing that was aged in 1.0 M NaOH solution for 40 hours, (c) CP/$Ni(OH)_2$ after OER tests, and (d) CP/$Ni(OH)_2$-ageing after OER tests.

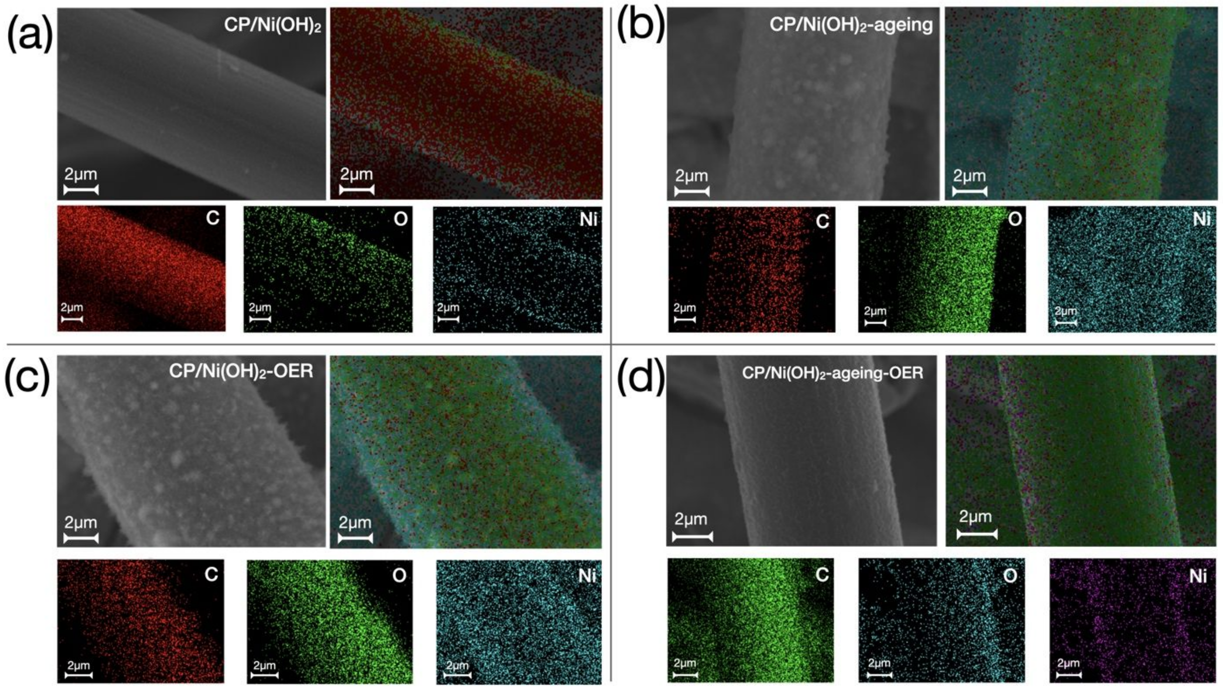

Fig. S10 EDX maps of (a) the as-prepared $Ni(OH)_2$ on carbon paper (CP) fibers, (b) CP/$Ni(OH)_2$-ageing that was aged in 1.0 M NaOH solution for 40 hours, (c) CP/$Ni(OH)_2$ after OER tests, and (d) CP/$Ni(OH)_2$-ageing after OER tests.

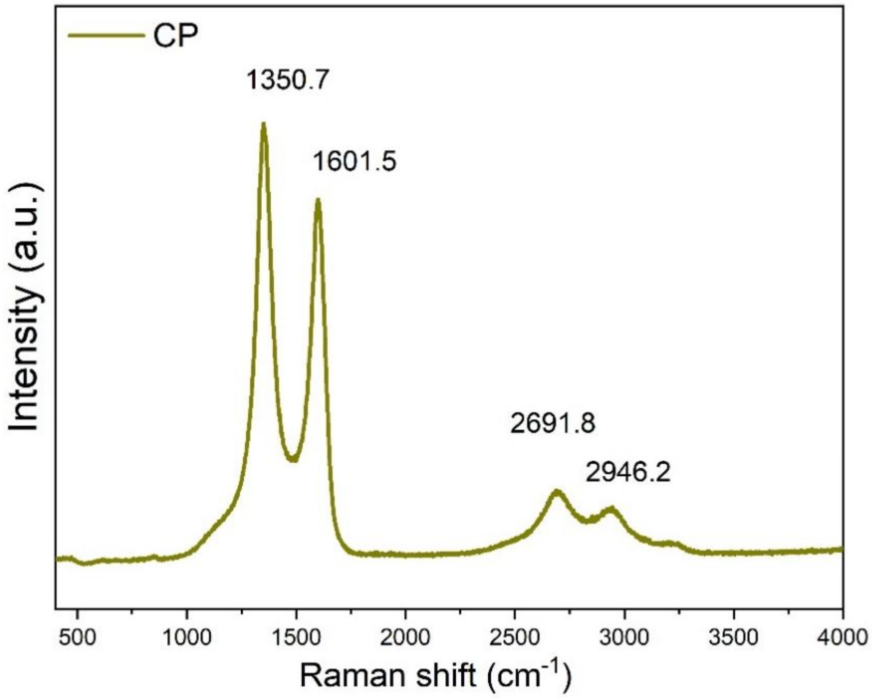

Fig. S11 Raman spectrum of the bare carbon paper.

Table S1. Comparison of the onset potential, photovoltage, photocurrent, and stability of the 3C-SiC/$Ni(OH)_2$-ageing photoanode (in this work) with the representative results of reported photoanodes under illumination of AM1.5G, 100mW/cm^2 in strongly alkaline solutions.

Photoanodes	Onset potential	**Photovoltage**	Current density (mA/cm^2) @1.23V_{RHE}	**Stability: Loss of the Initial Photocurrent J_{ph}**	Electrolyte	Ref
**3C-SiC/$Ni(OH)_2$-ageing**	**0.10 V_{RHE}**	**1.10 V**	**2.01**	**7% loss of J_{ph} after 10 hours**	**1M NaOH (pH =13.6)**	**This work**
Si/NiFeP	1.05 V_{RHE}	**0.43 V**	15.6	**42% loss of J_{ph} after 17 hours**	1M KOH	1
Si/$Co(OH)_2$	1.16 V_{RHE}	**0.49 V**	2.62	**77% loss of J_{ph} after 3 hours**	1M NaOH	2
Si/TiO_2/$Co(OH)_2$	1.16 V_{RHE}	**0.49 V**	2.61	**19% loss of J_{ph} after 4 hours**	1M NaOH	
Si/CoO_x (ALD)	1.03 V_{RHE}	**0.58 V**	23.4	**14% loss of J_{ph} after 2500 hours**	1M KOH	3
$BiVO_4$/CoO_x (ALD)	0.40 V_{RHE}	**0.83 V**	1.49	**50% loss of J_{ph} after 1 hour**	0.1M KOH	4
$BiVO_4$//$ZnFe_2O_4$	0.50 V_{RHE}	**NA**	2.82	**40% loss of J_{ph} after 3000 seconds**	0.1M KOH	5
$BiVO_4$/phenylenepyromellitimide	0.60 V_{RHE}	**NA**	5.48	**70% loss of J_{ph} after 6 hours**	0.1M phosphate (pH = 12)	6
Fe_2O_3 hollow-sphere-Array Photoanode	0.90 V_{RHE}	**NA**	2.26	**15% loss of J_{ph} after 10 hours**	1M KOH	7
Fe_2O_3/$NiFeO_x$	0.45 V_{RHE}	**0.8 V**	1.28	**Negligible loss of J_{ph} after 10 hours**	1M NaOH	8
Fe_2O_3/Ni-carbon nanosheets	0.80 V_{RHE}	**NA**	1.85	**10% loss of J_{ph} after 15 hours**	1M KOH	9
Grey Fe_2O_3	0.61 V_{RHE}	**0.41 V**	1.43	**Negligible loss of J_{ph} after 80000 seconds**	1M NaOH	10
GaAs/TiO_2/Ni core-shell nanorods	0.35 V_{RHE}	**NA**	12.6	**16% loss of J_{ph} after 1 hour**	1M NaOH	11
GaAs/TiO_2/Ni nanorods	0.50 V_{RHE}	**NA**	10.9	**56% loss of J_{ph} after 1 hour**	1M NaOH	
GaAs/TiO_2	0.58 V_{RHE}	**NA**	8.75	**62% loss of J_{ph} after 1 hour**	1M NaOH	

$GaP/TiO_2/Ni$	0.90 V_{RHE}	**0.59 V**	14.5	**8% loss of J_{ph} after 5 hours**	1M KOH	12
$In:GaN/Ta_3N_5/Mg:GaN/NiCoFe\text{-}B_i$	0.38 V_{RHE}	**NA**	9.32	**20% loss of J_{ph} after 10 hours**	1M KOH	13
$GaN/Ta_3N_5/FeNiCoO_x$ nanorods	0.75 V_{RHE}	**NA**	10.8	**8% loss of J_{ph} after 45 minutes**	1M KOH	14
$GaN/Ta_3N_5/NiFeO_x$	0.60 V_{RHE}	**NA**	7.23	**48% loss of J_{ph} after 80 mintues**	1M KOH	15

Table S2. Fitting values of EIS data of 3C-SiC, 3C-SiC/$Ni(OH)_2$ and 3C-SiC/$Ni(OH)_2$-ageing under AM1.5G 100 mW/cm^2 illumination. The EIS data were fitted with the equivalent circuit shown in the inset of Figure 4d, which consists of the series resistance (R_s), charge-transfer resistance (R_{ct}) from the semiconductor bulk to its surface, the constant phase element of the space-charge capacitance (CPE_{SC}), the charge-transfer resistance across the photoanode/electrolyte interface ($R_{ct, trap}$) and the corresponding capacitance (CPE_{trap}).

	**3C-SiC**	**3C/$Ni(OH)_2$**	**3C/$Ni(OH)_2$-ageing**
R_s (Ω cm^2)	15.6	16.2	3.81
R_{ct} (Ω cm^2)	594	649	161
CPE_{SC}-T	2.70×10^{-7}	1.00×10^{-6}	1.02×10^{-6}
CPE_{SC}-P	0.87	0.58	0.51
$R_{ct, trap}$ (Ω cm^2)	14350	1370	247
CPE_{trap}-T	2.00×10^{-6}	8.05×10^{-6}	5.62×10^{-5}
CPE_{trap}-P	0.86	0.58	0.46

Reference

1. F. Li, Y. Li, Q. Zhuo, D. Zhou, Y. Zhao, Z. Zhao, X. Wu, Y. Shan and L. Sun, *ACS Appl. Mater. Interfaces,* 2020, **12**, 11479-11488.
2. T. Yu, Z. Zhang, X. Yin, A. Kvit, Q. Liao, Z. Kang, X. Yan, Y. Zhang and X. Wang, *Nat. Energy*, 2017, **2**, 1-7.
3. X. Zhou, R. Liu, K. Sun, K. M. Papadantonakis, B. S. Brunschwig and N. S. Lewis, *Energy Environ. Sci.,* 2016, **9**, 892–897.
4. M. F. Lichterman, M. R. Shaner, S. G. Handler, B. S. Brunschwig, H. B. Gray, N. S. Lewis and J. M. Spurgeon, *J. Phys. Chem. Lett.*, 2013, **4**, 4188.
5. T. W. Kim and K. S. Choi, *J. Phys. Chem. Lett.*, 2016, **7**, 447–451.
6. B. Gao, T. Wang, Y. Li, Y. Guo, H. Xue, J. He and Y. Zhao, *J. Mater. Chem. A*, 2021, **9**, 3309–3313.
7. R. Yang, S. Xiao, J. Zhang, S. Tang, R. Xu and Y. Tong, *Small*, 2024, **20**, 2310752.
8. J. W. Jang, C. Du, Y. Ye, Y. Lin, X. Yao, J. Thorne, E. Liu, G. McMahon, J. Zhu, A. Javey, J. Guo and D. Wang, *Nat. Commun.*, 2015, **6**, 7447.
9. G. Yang, Y. Li, H. Lin, X. Ren, D. Philo, Q. Wang, Y. He, F. Ichihara, S. Luo, S. Wang and J. Ye, *Small Methods*, 2020, **4**, 2000577.
10. P. F. Liu, C. Wang, Y. Wang, Y. Li, B. Zhang, L. R. Zheng, Z. Jiang, H. Zhao and H. G. Yang, *Sci. Bull.*, 2021, **66**, 1013-1021.
11. M. Alqahtani, A. Kafizas, S. Sathasivam, M. Ebaid, F. Cui, A. Alyamani, H. H. Jeong, T. C. Lee, P. Fischer, I. Parkin, M. Grätzel and J. Wu, *ChemSusChem*, 2020, **13**, 6028–6036.
12. S. Hu, M. R. Shaner, J. A. Beardslee, M. F. Lichterman, B. S. Brunschwig, N. S. Lewis, *Science*, 2014, **344**, 1005–9.
13. J. Fu, Z. Fan, M. Nakabayashi, H. Ju, N. Pastukhova, Y. Xiao, C. Feng, N. Shibata, K. Domen and Y. Li, *Nat. Commun.*, 2022, **13**, 729.
14. Y. Pihosh, V. Nandal, T. Higashi, R. Shoji, R. Bekarevich, H. Nishiyama, T. Yamada, V. Nicolosi, T. Hisatomi, H. Matsuzaki, K. Seki and K. Domen, *Adv. Energy Mater.*, 2023, **13**, 2301327.
15. T. Higashi, H. Nishiyama, V. Nandal, Y. Pihosh, Y. Kawase, R. Shoji, M. Nakabayashi, Y. Sasaki, N. Shibata, H. Matsuzaki, K. Seki, K. Takanabe and K. Domen, *Energy Environ. Sci.*, 2022, **15**, 4761-4775.
